# Sir William J. Sinclair

**Published:** 1912-09-14

**Authors:** 


Sir William J. Sinclair, Professor of Obstetrics and Gynæcology at Victoria University, died last week at the age of sixty-six. Sir William was educated at Aberdeen University, where he qualified in 1873. Thence he went to be resident medical officer at St. Mary's Hospital, Manchester, to which he afterwards became physician. In the meanwhile he studied obstetrics in Vienna, after which he returned to Manchester. The list of his appointments and contributions to medical literature is a lengthy one; perhaps the most notable of his literary activities were his editorship of the *Medical Chronicle* and the establishment ten years ago of the *Journal of Obstetrics and Gynæcology of the British Empire.* Sir William Sinclair was knighted in 1904.

